# Transcriptome display during tilapia sex determination and differentiation as revealed by RNA-Seq analysis

**DOI:** 10.1186/s12864-018-4756-0

**Published:** 2018-05-15

**Authors:** Wenjing Tao, Jinlin Chen, Dejie Tan, Jing Yang, Lina Sun, Jing Wei, Matthew A. Conte, Thomas D. Kocher, Deshou Wang

**Affiliations:** 1 Key Laboratory of Freshwater Fish Reproduction and Development (Ministry of Education), Key Laboratory of Aquatic Science of Chongqing, School of Life Sciences, Southwest University, Chongqing, 400715 China; 2 Department of Biology, University of Maryland, College Park, MD USA

**Keywords:** Sex determination and differentiation, Tilapia, RNA-seq analysis

## Abstract

**Background:**

The factors determining sex in teleosts are diverse. Great efforts have been made to characterize the underlying genetic network in various species. However, only seven master sex-determining genes have been identified in teleosts. While the function of a few genes involved in sex determination and differentiation has been studied, we are far from fully understanding how genes interact to coordinate in this process.

**Results:**

To enable systematic insights into fish sexual differentiation, we generated a dynamic co-expression network from tilapia gonadal transcriptomes at 5, 20, 30, 40, 90, and 180 dah (days after hatching), plus 45 and 90 dat (days after treatment) and linked gene expression profiles to both development and sexual differentiation. Transcriptomic profiles of female and male gonads at 5 and 20 dah exhibited high similarities except for a small number of genes that were involved in sex determination, while drastic changes were observed from 90 to 180 dah, with a group of differently expressed genes which were involved in gonadal differentiation and gametogenesis. Weighted gene correlation network analysis identified changes in the expression of *Borealin, Gtsf1, tesk1, Zar1*, *Cdn15*, and *Rpl* that were correlated with the expression of genes previously known to be involved in sex differentiation, such as *Foxl2*, *Cyp19a1a*, *Gsdf*, *Dmrt1*, and *Amh*.

**Conclusions:**

Global gonadal gene expression kinetics during sex determination and differentiation have been extensively profiled in tilapia. These findings provide insights into the genetic framework underlying sex determination and sexual differentiation, and expand our current understanding of developmental pathways during teleost sex determination.

**Electronic supplementary material:**

The online version of this article (10.1186/s12864-018-4756-0) contains supplementary material, which is available to authorized users.

## Background

Sex-determination mechanisms in mammals and birds are highly conserved. The *Sry* gene, which arose by gene duplication 170 million years ago (Mya), determines male sex in most mammals [1]. Analysis of human and mouse mutants has identified many additional genes involved in sexual differentiation, including *Dmrt1*, *Foxl2*, *Rspo1*, *Sox9*, and *Wnt4* [2, 3]. Some of these same genes have been implicated as sex determiners in other tetrapods. Most birds share a ZW system of sex determination that arose 130 Mya, which is mediated at least in part by expression of *Dmrt1* [4]. A duplicated and truncated *Dmrt1* gene on the female-specific W chromosome, *Dmw*, is required for female sex determination in *Xenopus laevis* [5, 6]. The fact that many of the same genes are involved in sex differentiation in these distantly related species suggests that the gene regulatory network underlying sex determination and sexual differentiation might be fundamentally similar among all tetrapods.

In contrast, teleost fishes are remarkably diverse in their sex chromosome systems [7–9]. Seven master sex-determining genes have been identified in teleosts, namely *dmy*/*dmrt1b*^*Y*^ in *Oryzias latipes* and *Oryzias curvinotus* [10, 11], *gsdf*^*Y*^ in *Oryzias luzonensis* [12], *sox3* in *Oryzias dancena* [13], *amhy* in *Odontesthes hatcheri* [14] and *Oreochromis niloticus* [15], *amhrII* in *Takifugu rubripes* [16], *gdf6Y* in *Nothobranchius furzeri* [17] and *sd*^Y^ in *Oncorhynchus mykiss* and several other salmonids [18, 19]. Moreover, sex chromosomes and sex determining genes of the same species were found to be diverse. For example, a tandem duplicate of *Amh* with a missense SNP on LG23 may contribute to male sex determination in some strains [15, 20], while main sex determining region of other strains has been found on LG1 [21]. The diversity of sex determining genes suggest there are at least some differences between mammals and teleosts in the structure of the gene network controlling sex determination. Indeed, *Gsdf* is found only in teleosts, and the teleost genomes lack *Fgf9,* suggesting that the specific function, regulation, and interconnection of the sex network has changed during the evolution of teleosts. Nevertheless, most of the genes *Foxl2*, *Dmrt1*, and pathways such as *Rspo1/Wnt/*TGF-β which are important in mammalian sex determination, still play key roles in teleost sex determination [22].

Research in *Drosophila* and *Caenorhabditis* identified linear pathways of gene interaction leading to sex determination. These pathways are thought to have evolved largely by addition of upstream master controllers, although the pathways may also be vulnerable to takeover at intermediate steps [23, 24]. In contrast, vertebrate pathways have been characterized as balanced networks, in which changes in any of a number of genes can shift the fate of the undifferentiated gonad [25, 26]. The developmental pathways following the initiation of sex determination in fishes may consist of a conserved cascade of genes regulated by sex steroid hormones [27]. Data from natural and induced sex reversal after primary sex determination in various fishes also suggest that sex is not a stepwise, hierarchical trait, but instead a consensus of interconnected gene networks that also incorporate environmental factors [28–30].

There are two general approaches to characterize the gene network underlying sex determination and differentiation in a particular evolutionary lineage. The first is to genetically map and identify the top-level sex determiner in each of several species. This is essentially a ‘natural mutant screen’ in which evolution has identified various genes affecting the underlying regulatory network. This approach has been particularly successful in medaka [10] and tilapia [15]. The second approach is systems biology. Sex-related genes frequently exhibit sexually dimorphic patterns of expression in the developing gonad both before and after overt differentiation of the testis or ovary. If we collect transcriptome data from male and female gonads at various developmental stages, we can use statistical methods to infer the regulatory network from the correlations of gene expression across samples. These two approaches are not mutually exclusive. Gonadal transcriptomic data with different top-level sex determiners might be combined to understand their shared gene regulatory network. Together, these approaches have identified several genes in the pathway of sex determination and sexual differentiation, but our understanding of it is far from complete. Gene co-expression analysis, uses the correlation (or related measures) of gene expression profiles across multiple samples to identify common patterns of regulation [31]. The most widely used method is Weighted Gene Co-expression Network Analysis (WGCNA) [32]. This method discovers groups of genes with similar patterns of gene expression, called modules. Regulatory networks and candidate genes associated with gonad differentiation have been identified using WGCNA in turbot and catfish [33, 34].

Tilapia is the world’s second most farmed fish, and an important source of animal protein around the world. Male tilapias grow faster than females, so the tilapia industry prefers to grow all-male populations. A better understanding of the molecular mechanisms of sex determination and differentiation will facilitate the production of mono-sex progeny for commercial production. The objectives of this study were to explore the molecular mechanism of tilapia sex determination and differentiation and to identify new genes involved in this process. Our approach was to collect a set of gonadal transcriptomes from genetic females and males, and sex-reversed individuals, through the course of gonad development from 5 to 180 days after hatch (dah). WGCNA was used to identify gene co-expression modules that are differentiated between ovary and testis during gonad development. Network neighbors of some canonical genes involved in vertebrate sex differentiation, including *Cyp19a1a*, *Foxl2*, *Dmrt1*, *Gsdf*, *3beta-Hsd*, *Cyp11b2*, and *Amh* were identified. Our ultimate goal was to identify putative regulatory links that might be tested by CRISPR modifications.

## Methods

### Fish materials and ethics statement

The founder strain of the Nile tilapia, firstly introduced from Egypt in Africa, was obtained from Prof. Nagahama (Laboratory of Reproductive Biology, National Institute for Basic Biology, Okazaki, Japan). Nile tilapia were maintained and reared in re-circulating aerated freshwater tanks at 26 °C prior to use. The monosex genetic female (XX) and male (XY) tilapia were obtained as described previously [20]. All fish experiments were conducted in accordance with the regulations of the Guide for Care and Use of Laboratory Animals and were approved by the Committee of Laboratory Animal Experimentation at Southwest University.

### Gonadal histology, RNA extraction and Illumina library preparation

All fishes were euthanized using an overdose of MS222 and stored in 70% EtOH. Special care was taken to minimize suffering of fish. Gonads were dissected at 5, 20, 30, 40, 90, and 180 dah, fixed in Bouin’s solution for 24 h at room temperature, dehydrated, and embedded in paraffin. All tissue blocks were sectioned at 5 μm and stained with hematoxylin and eosin.

A total of 300, 200, 150, and 120 gonads for each sex were pooled at 5, 20, 30, and 40 dah, respectively. Total RNA was extracted from gonads of XX and XY fishes using the Trizol Reagent (Invitrogen, Carlsbad, CA) according to the manufacturer’s instruction, and purified with DNaseI (RNase-free 5 U/uL) to eliminate genomic DNA contamination. All the tilapia gonadal samples used in sequencing were again genetically sexed using a marker tightly linked to the AMH polymorphism on LG23 [15]. Both agarose gel electrophoresis and a Nanodrop spectrophotometer were used to assess the integrity and concentration of RNA. Poly-T Oligo-attached magnetic beads were used to isolate poly(A) mRNA from total RNA. The first and second strand cDNA were synthesized form the fragmented poly(A) mRNA according to the Illumina sample preparation guide. Sequencing was performed on the HiSeq system. The reads have been deposited in NCBI’s Sequence Read Archive (SRA) database with accession number SUB3254083. Gonadal transcriptomes of normal development at 90 and 180 dah were downloaded from SRA database [35]. Gonadal transcriptomes of 45 dat (days after treatment) and 90 dat were from Fadrozole-treated XX fishes started at 90 dah [28]. These 90 dah-old XX fish were fed with a diet sprayed with 95% ethanol containing Fadrozole (Sigma) at a concentration of 200 μg/g diet for 45 (45 dat) and 90 days (90 dat).

### Gonadal transcriptomes

After trimming adapters and low quality sequence with Trimmomatic [36], the clean reads were aligned to a tilapia reference genome assembly [37] using Tophat [38], allowing up to 2 base mismatches per read. NCBI RefSeq annotations were used to guide the Cufflinks assembly. Cuffdiff and Cuffnorm [39] were used to determine gene expression. A threshold of FPKM > 1 in at least one sample was used to filter the extremely low expression gene. Clustering of samples by global gene expression data (transformed to log_2_FPKM) was constructed using the heatmap function of Gplot in R. A false discovery rate (FDR) < 0.05 and two-fold difference in expression between XX and XY gonads at the same stage were used as thresholds to identify differentially expressed genes (DEGs).

### Gene co-expression network analysis

To further understand the relationships between genes, a network analysis based on gene-to-gene correlations was performed using WGCNA, an R package [32]. The automatic one-step network construction and module detection method was used, which include a signed topological overlap matrix (TOM), a softPower of 18, a minimal module size of 30, and a branch merge cut height of 0.19. The module eigengene (the first principal component of a given module) was calculated and used to test the association of modules with “Genotype”, “Phenotype”, “Developmental stages”, and “key genes (*Cyp19a1a*, *Foxl2*, *Dmrt1*, *Gsdf*, *3beta-Hsd*, *Cyp11b2*, and *Amh*)” of 16 samples. A value of 0 was assigned to sex reversal samples, a value of 1 to female samples and a value of − 1 to male samples. Gene significance (GS, the correlation between gene expression and traits), total network connectivity, and module membership (also known as eigengene-based intramodular connectivity), were calculated for all modules. The most relevant connections with key genes (*Cyp19a1a*, *Foxl2*, *Dmrt1*, *Gsdf*, *3beta-Hsd*, *Cyp11b2*, and *Amh*), using a high threshold power and differential expression in at least 4 stages, were kept as the candidate genes.

### Data validation by in situ hybridization (ISH)

To validate which population of cells in the developing gonads express particular genes, we performed ISH on ovaries and testes from adult tilapia (180 dah) as described previously [40]. Gonads were dissected and fixed in 4% paraformaldehyde in 0.1 M phosphate buffer (pH 7.4, 4% PFA) at 4 °C overnight. After fixation, the tissues were embedded in paraffin and cross sections were cut at 5 μm. Digoxigenin (DIG)-labeled sense and antisense RNA probes were transcribed in vitro from linearized pGEM-T easy plasmids containing-*Borealin*, *Gtsf1*, *Zar1*, *C15orf65* or *Rbp2* cDNA using a RNA labeling kit (Roche).

## Results and discussion

### Timeline of gonad development

**Fig. 1:**
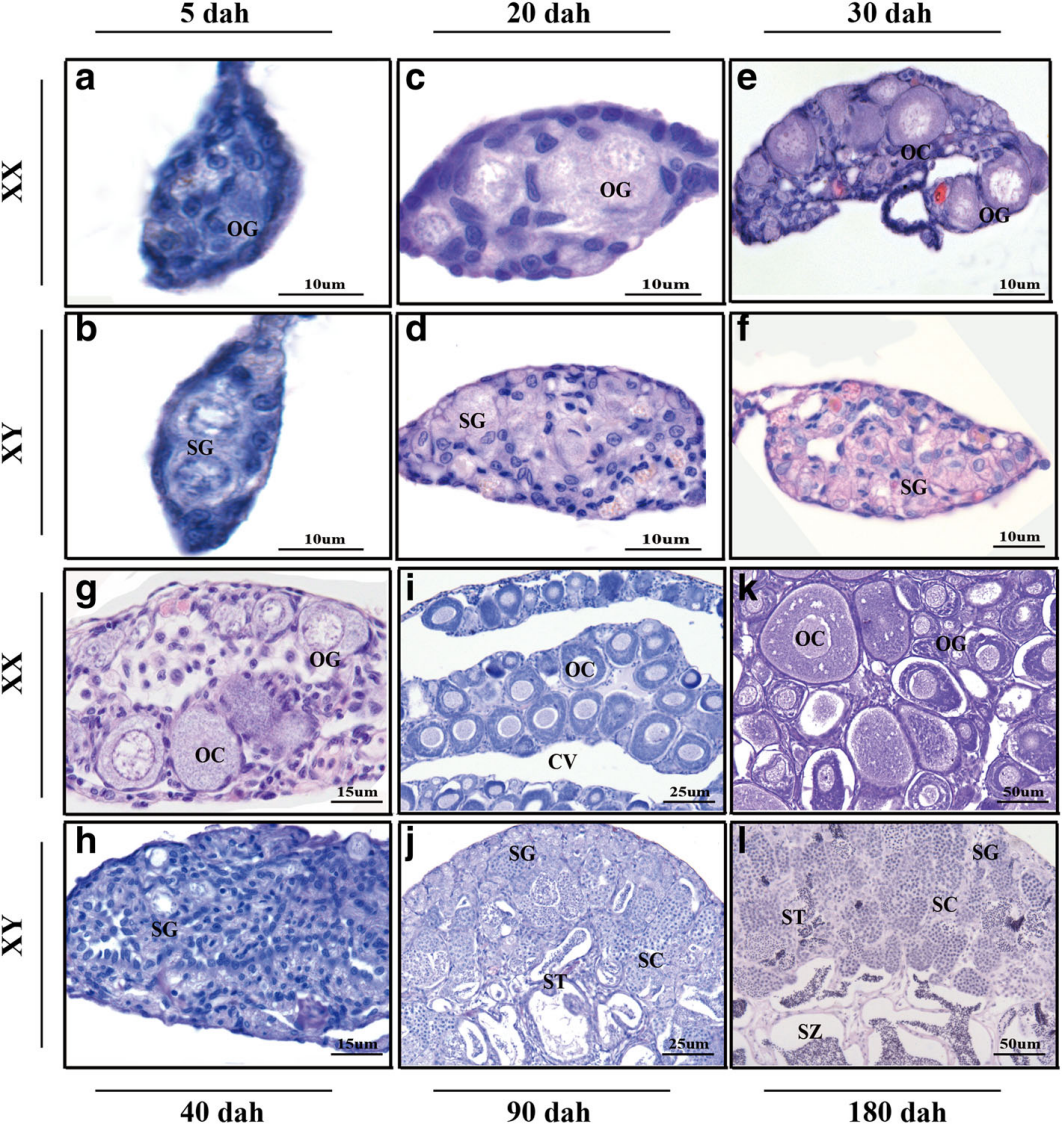
Histological analysis of characteristic gonadal stages in tilapia ovary (XX) and testsis (XY). Panels indicate gonads sampled from fish at different days after hatch (dah) as follows: **a** and **b** at 5 dah, **c** and **d** at 20 dah, **e** and **f** at 30 dah, **g** and **h** at 40 dah, **i** and **j** at 90 dah, and **k** and **l** at 180 dah. OG, oogonia; SG, spermatogonia; OC, oocytes; CV, ovarian cavity; ST, spermatids; SC, spermatocytes

Histological staining can be used to observe morphological differentiation during the development of tilapia gonads [41]. In this study, we focused on samples beginning at the earliest stage gonads can be dissected (5 dah) through to sexual maturation (180 dah). Histological observations were made of the XX and XY gonads at 5, 20, 30, 40, 90, and 180 dah. Sex determination occurs during the earliest stages of development, when the fate of bi-potential gonadal primordium is directed toward becoming a testis or an ovary. Typically, there is no overt morphological differentiation during the period of sex determination. Between 5 and 20 dah, no morphological differences could be observed in the germ cells of XX and XY gonads (Fig. 1a, b, c, and d). Only a handful of genes show transcriptional differences between male and female gonads at 5 dah. During the period from 5 to 20 dah, the primordial gonad loses its intersexuality and the phenotypic sex is fixed as in other gonochoristic fish [42]. Sexual differentiation begins between 30 and 40 dah, as demonstrated by the initiation of meiosis with appearance of the oocytes. At this stage, the germ cells in the testis have not initiated meiosis (Fig. 1e, f, g, and h). At 90 dah, spermatogenesis began in testis, while an ovarian cavity was observed in the females (Fig. 1i and j). At 180 dah, females develop a mature ovary; in its swollen anterior end, large previtellogenic oocytes are present in a lamellar structure. At the same time, males develop a mature testis characterized by the appearance of germ cell meiosis and numerous spermatozoa in the efferent duct (Fig. 1k and l).

### Gonadal transcriptome sequencing of tilapia gonad

To generate a comprehensive profile of the tilapia gonadal transcriptome during sex determination and differentiation, we sampled XX and XY gonads at 5, 20, 30, 40, 90, and 180 dah. Transcriptomes were sequenced to a depth of ~ 90 million reads after trimming, and 78% of these were mapped to the tilapia genome assembly. Of these, 90% reads were mapped to annotated regions and 96.4% of the mapped reads were placed uniquely, indicating the good quality of reads. Overall, 28,586 transcripts were expressed in gonads during at least one of the developmental stages. A threshold FPKM > 1 in at least one sample was used to define genes as robustly expressed [43, 44]. In total, 19,188 genes were retained for subsequent analysis. At later stages of development (40 dah, 90 dah, and 180 dah), thousands of genes were differentially expressed between XX and XY gonads. These genes are enriched for functional annotations related to transcription factor binding and nuclear hormone receptor binding.

### Global gene expression diverges at 30 dah

**Fig. 2:**
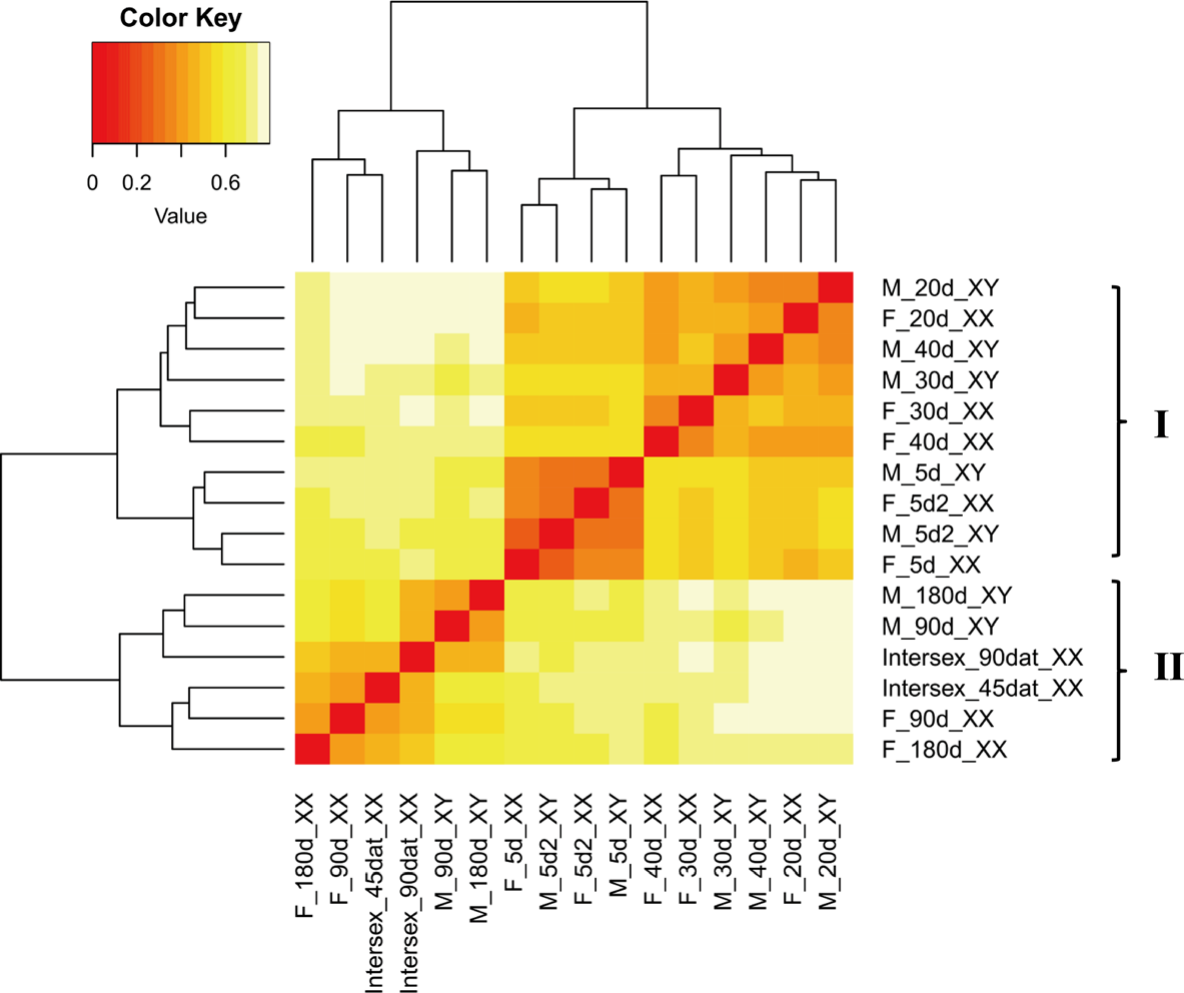
Global gene expression profiles in XX and XY gonads from 5 to 180 dah. Two different groups were clearly identified. One consisted of samples at earlier stages (Branch I: 5, 20, 30, and 40 dah), and the other consisted samples at later developmental stages (Branch II: 90, 180 dah). Intersex_45 dat XX and Intersex_90 dat were gonadal samples of 90dah XX fish treated with Fadrozole for 45 and 90 days, respectively [29]. F, female; M, male

The gene expression patterns of gonads at 5 (with two replicates), 20, 30, 40, 90, and 180 dah, as well as that of 45 and 90 dat, were hierarchically clustered in a heatmap using the Pearson correlation coefficient as distance measure (Fig. 2). Two different groups were clearly identified. One consisted of fishes at earlier stages (Branch I: 5, 20, 30, and 40 dah), and the other consisted fishes at later developmental stages (Branch II: 90, 180 dah). In Branch I, XX and XY gonads are clustered together at both 5 and 20 dah. The first molecular signs of sex differentiation were observed at 5 dah, characterized by increased expression of *Dmrt1* in the testis, and increased expression of *Cyp19a1a* in the ovary, as reported in previous studies [35, 42]. These genes are an indication of some of the earliest steps in the female and male cascade during gonadal sex determination in tilapia. However, most other genes showed no obvious differences between males and females at 20 dah. Global gene expression of male and female gonads started to differentiate at 30 dah, the period when morphological differentiation of the gonads is first observed. Gonadal samples at later stages consistently clustered into two groups corresponding to the XX and XY genotypes. Gene expression of 45 dat sample grouped with that of XX gonads at 90 and 180 dah, while gene expression of 90 dat sample clustered with that of XY gonads at 90 and 180 dah.

### Network analysis and association of sex candidate genes with modules

**Fig. 3:**
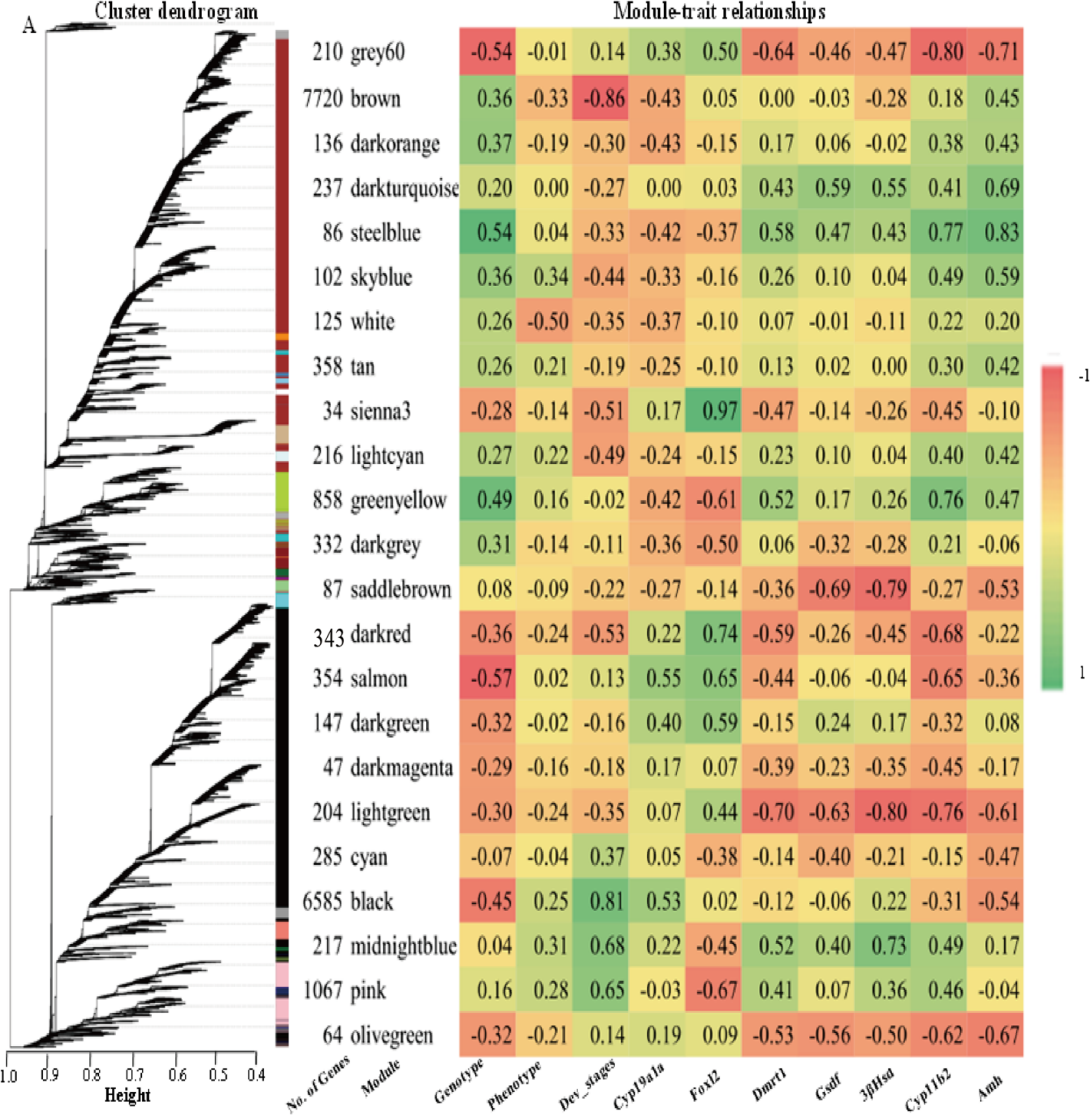
Gene expression modules constructed by the WGCNA. **a** the clustering dendrogram of genes expressed in the XX and XY gonads is shown. WGCNA identifies gene modules using the cutreeDynamic function, which detects clusters in a dendrogram depending on their shape [32]. Original modules of very similar coexpression are merged. Color name-based module labels are generated automatically by WGCNA and are shown along with the number of genes belonging to each module. **b** Association between modules and traits (such as Genotype, Phenotype, Developmental stages, and Key genes) is depicted. Rows correspond to module eigengenes (shown on the left with the colored boxes), columns to a trait (indicated below each column). Each cell contains the *P* value of the trait-module association. Large positive values indicate a strong correlation, while large negative values indicate a strong negative association

Regulatory networks can be organized into modules of highly interconnected genes, which are implicated in the same biological processes or associated with particular cellular states. Gene co-expression analysis is based on the similarity of gene expression profiles across multiple samples or conditions. In general, adding more datasets obtained from different samples increases the predictive power of this method [45]. In the present study, we performed a network analysis based on 16 samples using WGCNA, which partitions a gene set into modules defined as branches of the co-expressed gene cluster tree. Our analysis resulted in 23 modules (Fig. 3) with sizes ranging from 37 to 7720 genes. For purposes of discussion, each of these modules is assigned a color. Unassigned genes might belong to smaller expression pathways because of the chosen minimal module size of 30 genes. Next, we used ANOVA to test for associations between the WGCNA modules and genotype, phenotype, developmental stages (dah), and the expression of key genes in vertebrate sexual development. Investigation of the relationships between the module eigengenes and traits uncovered correlation coefficients that varied widely from -0.57 to 0.54 with genotype and from − 0.86 to 0.81 with developmental stage. The black and brown modules were significantly associated with the developmental stage, and grouped more than 72% of the genes included in the analysis. This result reflected the dramatic changes in gene expression associated with gonadal morphogenesis. Most of the genes already known to be involved in sex differentiation were assigned to these two large modules, including *Cyp19a1a*, *AmhrII*, *Sox9b*, *Fst*, *Sox3*, *Gata5*, and *Lim1*. Several genes in the hedgehog (HH) signaling pathway, including *Shh*, *Ihh*, and *Ptch2,* were also placed in brown and black modules.

Genes within a module are frequently known to be functionally related. For instance, *Piwil1* and *Sox30* from the midnight module are both involved in mRNA binding and regulation of translation [46, 47]. Similarly, another study has provided evidence to suggest that *Piwil1* was expressed with a proposed regulatory role in striped bass [48]. The tan module included genes involved in epigenetic regulation throughout gonad development, including histones and methyltransferase. Some homologs, such as *Ara* and *Arb*, *Sox9a* and *Sox9b* are in the same module. However, other homologous genes, *Cyp19a1a* and *Cyp19a1b*, *Sox8a* and *Sox8b* were placed in different modules, suggesting a functional difference of these duplicate genes. The modules steelblue and darkturquoise were associated with canonical genes reported to be important in testis development, including *Amh*, *Cyp11b2*, *Dmrt1*, *Gsdf*, and *3beta-Hsd*. Other genes in these modules, such as *Tex2*, *Er2*, *Fgfr1*, *Ar*, and *Cyp2k1*, were known to be key regulators of testis differentiation. Genes in the pink module were also preferentially expressed in males, especially at the later stages (90 and 180 dah), suggesting their crucial roles in spermatogenesis. For example, null *Klhl10* mice causes haploinsufficiency with meiotic arrest, absence of mature spermatozoa in semen and male infertility [49]. *Spata6* was essential for the connecting piece formation and tight head–tail conjunction during spermiogenesis in human [50]. The pink module also includes *Tdrd6* and *Hira*, which are involved in transcription regulation via modification of histones [51, 52]. Most of the genes in the smallest module sienna3, including gene *Hsp11* and *Svep1,* were overexpressed in the ovaries and significantly associated with *Foxl2*.

### Apoptosis associated with the masculinization of the female gonad

The effects of Fadrozole (aromatase inhibitor) on gene expression during sex differentiation have been investigated in several teleost species, including zebrafish [53], rainbowfish [54], tilapia [28], and pejerrey [55]. Regardless of the underlying differences in sex determining mechanism, a shared characteristic of these species is that apoptosis of somatic cells in the gonads plays a key role during testicular differentiation. Degeneration of oocytes, together with the suppression of female pathway genes and activation of male pathway genes, was observed during Fadrozole-induced sex reversal of tilapia [28]. In the present study, module white had the most significant correlation with the trait of phenotype (*r* = − 0.5). Genes in this module were enriched in functional annotations relevant to PPAR signaling pathwayand oxidoreductase activity. The expression of apoptosis-involved genes precedes the development of testicular structures and degeneration of oocytes in fadrozole-treated fish. These apoptosis-related processes likely play a role in the transdifferentiation of the gonads during sex reversal, as already known from other fishes [56, 57].

### Candidate genes involved in tilapia sex differentiation

**Fig. 4:**
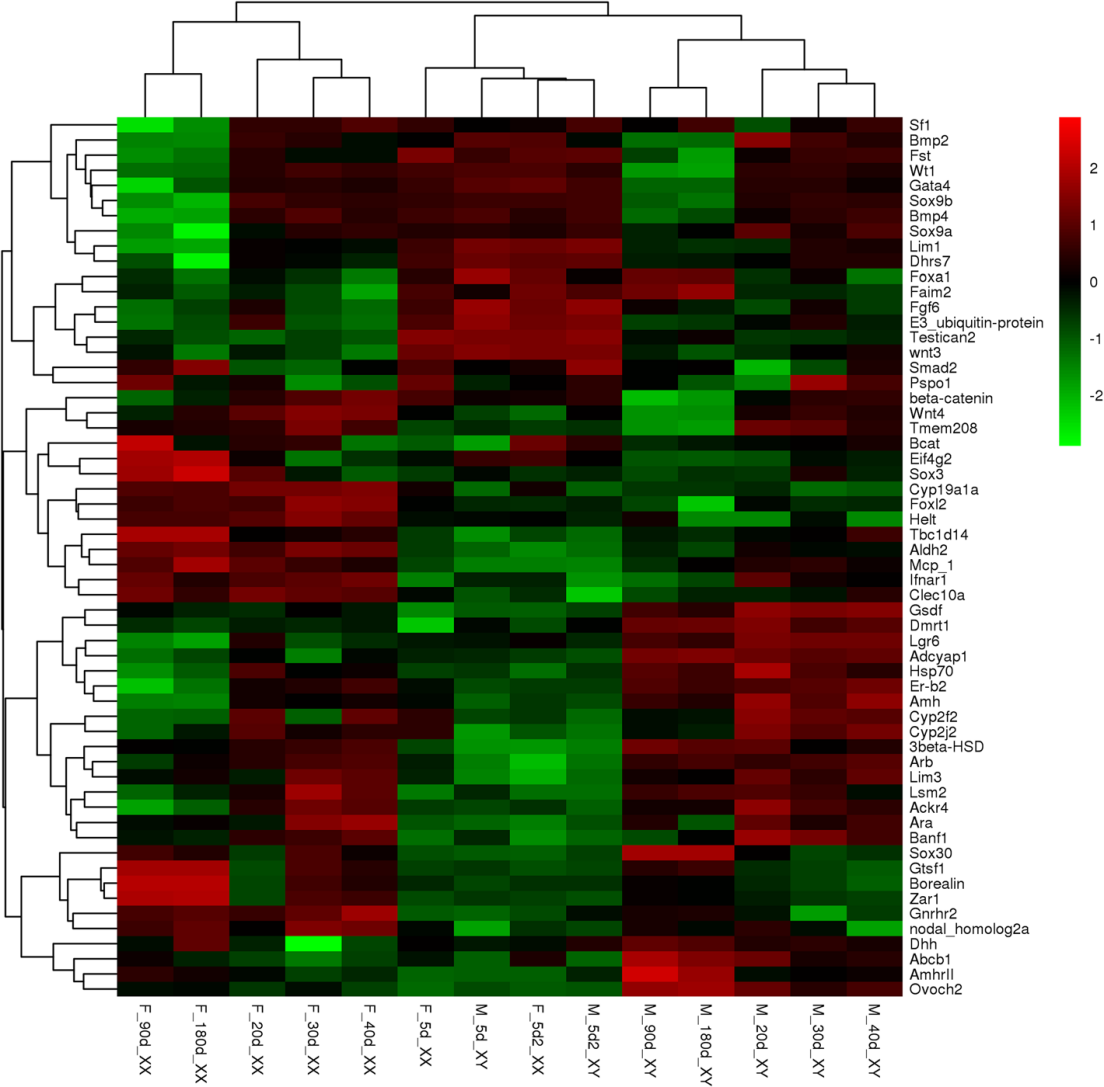
Heatmap analysis of key genes involved in tilapia sex determination and differentiation. Each row represents a gene listed on the right. Each column stands for a gonadal sample specified in Fig. 1. The expression of genes is color coded from low (green) to high (red). Full gene names are shown in Additional file 1

The top-level genes controlling sex determination vary among vertebrate species, but the downstream genes that control sexual differentiation appear to be relatively conserved [30]. To identify other candidate genes that might be involved in tilapia sex determination and differentiation, we identified sex-biased genes that also showed strong correlations to key genes (*Cyp19a1a*, *Foxl2*, *Dmrt1*, *Gsdf*, *Amh*, and *3beta-Hsd*) already known to be involved in these processes in other vertebrates (Additional file 1). A hierarchically-clustered heatmap of known sexual development genes and candidate genes showing sexually dimorphic expression in at least 4 developmental stages was constructed additionally to display the expression of these genes (Fig. 4). Interestingly, this subset of genes produced a different clustering result compared with that observed in the overall gene cluster. The gene expression patterns of XX gonads at 20, 30, 40, 90, and 180 dah were hierarchically clustered, while gene expression of XY gonads at these stages grouped together. Namely, these genes displayed obviously different sex-specific gene expression profiles as early as 20 dah. However, similar clustering pattern of Fig. 2 was found while we constructed heatmap using all the genes in Additional file 1, indicating most genes in this list were involved in tilapia sexual differentiation at later stages. Until now, no example of a sex determining gene is still highly expressed in adult fish. For example, the expression of the sex determiner *Amhy* in Nile tilapia was detected at 5 dah, increased at 20 dah and peaked at approximately 40 dah, and decreased at 90 and 180 dah [15]. Thus, further study of sex determiners expression covering the development from embryo to adults is needed to explore the gene regulatory network during early sex determination.

Knockout of *Foxl2* and *Cyp19a1a* resulted in 100% complete female to male sex reversal [58], while disruption of *AmhRII* and *Gsdf* resulted in male to female sex reversal with normal ovaries in tilapia [15, 59]. Besides these previously reported sex-determining genes, some new candidates genes identified in present study possibly play an important role in tilapia sex determination and sexual differentiation. For example, mutations of *Gtsf1* in *Drosophila* caused de-repression of transposons and loss of ovary follicle layers, resulting in female infertility [60]. *Zar1* displayed ovary-specific expression in mice [61], and it was up-regulated at XX tilapia gonad. Notably, proteomic analysis of striped bass ovary indicated that *Zar1* was also predominantly detected in early secondary and vitellogenic growth ovary (equivalent to phase I and phase II oocytes in tilapia) and protein expression dramatically decreased during post-vitellogenesis [52]. A recent study indicated that mutation of *Zar1* in zebrafish resulted in early oocyte apoptosis and fully penetrant male development, while the EE2-treated *Zar1* homozygous mutants were recovered as females [62].

Other genes were not sexually dimorphically expressed at 5 and 20 dah, but highly expressed at later stages. These genes, including *Pacap*, *Hsp70*, *Lgr6*, *Mphosph6*, *Svep1*, and *Tbxas1*, might act late in gonad differentiation. *Pacap*, also known as *Adcyap1*, is involved in gonadotropin synthesis and release, either alone or in cooperation with GnRH [63, 64]. The coexpression of *Pacap*, with *Cyp11b2*, *Tbxas1*, *Amh*, *Abcb11*, and *Lgr6* in tilapia testis, indicated its critical role in tilapia endocrine systems. *Hsp70* is associated with gonad development and egg quality in different species [64–68]. In the tilapia gonad, *Hsp70* was highly expressed in testis during gonad differentiation, and co-expressed with *Sox9a*, *Gata4*, and *Lhx6*. Consistently, a previous study indicated that *HSP70* was an interacting partner for SOX9 [67]. Thus, *Hsp70* is a good candidate for future studies at investigating sex-related regulatory network in tilapia and possibly other fish. The higher expression of *Lgr6* in tilapia testis*,* together with *Lgr4* and *Lgr5*, might encode orphan 7-transmembrane receptors and mediate Wnt/β-catenin and Wnt/PCP signaling in tilapia gonad differentiation, as indicated in mammals [69–71]. *Mphosph6* play a critical role in M-phase characteristics of growing oocytes in mouse [72]. In this study, *Mphosph6* was highly expressed in tilapia ovaries from 90 to 180 dah, indicating its its role in tilapia oocyte maturation. A previous study showed that both estradiol and TNFα regulated *Svep1* expression [73], but its function in sexual differentiation has not been reported yet. *Tbxas1*, also called *Cyp5*, was highly expressed in mouse testis and essential for testis development [74].

Finally, 17 uncharacterized transcripts were found to be differentially expressed in XX and XY gonads at various time points. Interestingly, 9 of these transcripts were identified as ncRNAs (LOC100696883, LOC102076355, LOC102077364, LOC102077676, LOC102078352, LOC102079282, LOC102080335, LOC102081303, LOC102082652). The presence of these ncRNAs may have significant consequences on tilapia sex determination as ncRNA (MHM) regulation of DMRT1 plays a pivotal role in *Gallus gallus* sex determination [75]. However, their functions and roles during tilapia sexual development need to be further investigated. This again confirmed that sex determination in vertebrates was far from being a linear pathway built from the bottom up; instead different pathways and multilayered feedback loops worked together in a non-hierarchical network to produce a male or female phenotype [76]. Both in vitro techniques (reporter assays) and in vivo (gene knockout) will be useful to evaluate such interactions and provide greater insight into the functional relationships between genes in this putative network.

### Validation of candidate genes by ISH

**Fig. 5:**
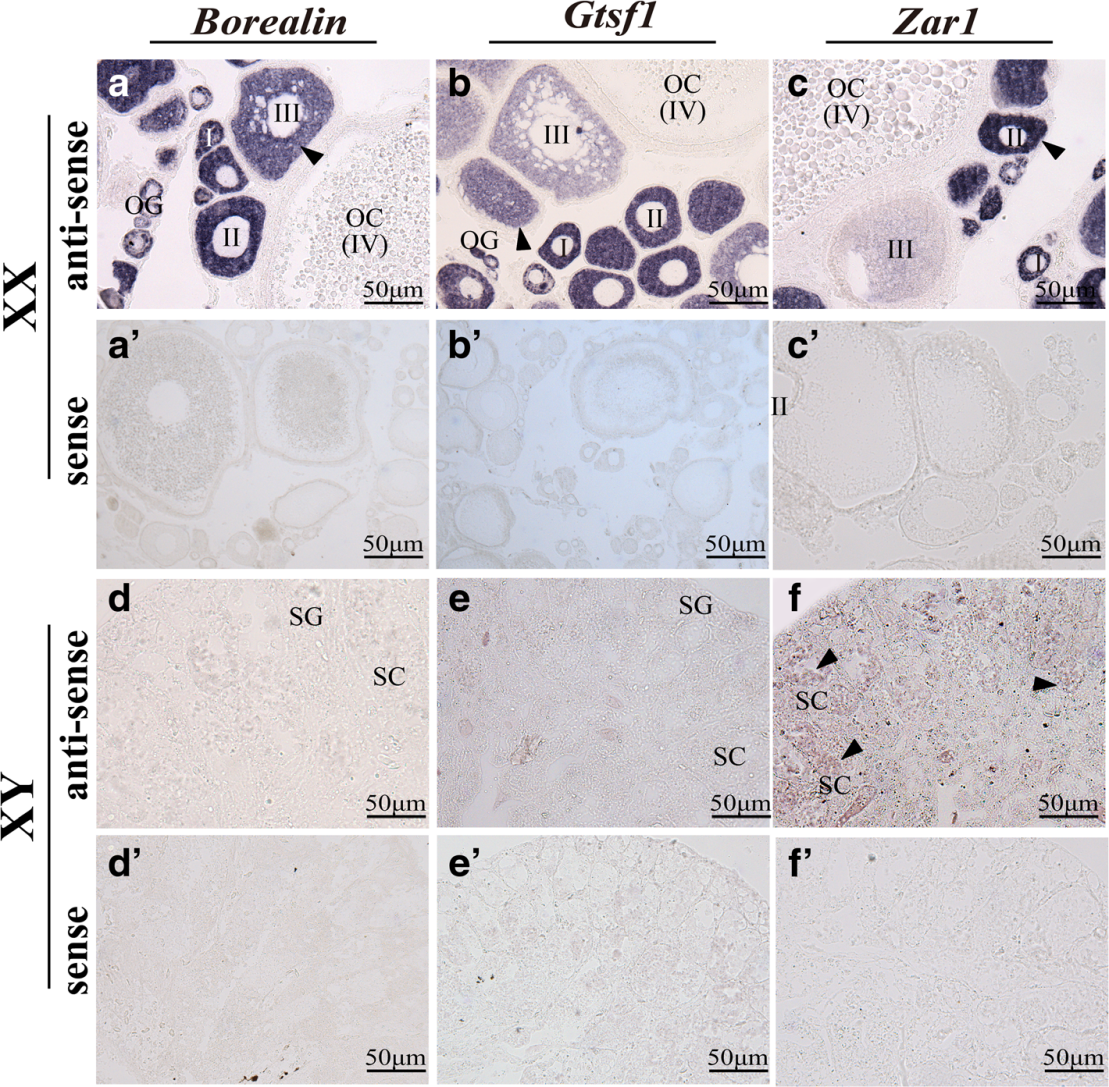
Cellular localization of *Borealin*, *Gtsf1* and *ZarI* in tilapia testis and ovary by ISH. *Borealin* was weakly expressed in the oogonia, highly expressed in the phase I and II oocytes of the ovary, but was not expressed in the phase III and IV oocytes (**a**), while no *Borealin* expression was found in the testis (**d**). *Gtsf1* was weakly expressed in the oogonia and phase I oocytes, highly expressed in the phase II oocytes, and moderately expressed in the phase II oocytes of the ovary (**b**), while no *Gtsf1* expression were detected in the testis (**e**). *ZarI* was highly expressed in the phase I and II oocytes, and weakly expressed in the phase III oocytes in the ovary (**c**), and moderately expressed in the spermatocytes in the testis (**f**). OG, oogonia; OC, oocytes; I-IV, phase I to phase IV oocytes; SG, spermatogonia; SC, spermatocytes; Arrowheads indicate the positive signal

The gonad is a mixture of different cell types. We used ISH to determine the cell types in which several of these new candidate genes, including *Borealin*, *Gtsf1* and *ZarI*, were expressed (Fig. 5). By ISH, *Borealin* was highly expressed in the phase I and II oocytes of the ovary. *Borealin* was moderately expressed in the phase III oocytes, but was not expressed in the phase IV oocytes. No *Borealin* expression was found in the testis. *Gtsf1* was weakly expressed in the oogonia and phase I oocytes, highly expressed in the phase II oocytes, and moderately expressed in the phase III oocytes of the ovary, while no *Gtsf1* expression were detected in the testis. *ZarI*, located on LG23, was highly expressed in the phase I and II oocytes, and weakly expressed in the phase III oocytes in the ovary, and moderately expressed in the spermatocytes in the testis. *Cdn15* and *Rpl* were expressed in XX and XY gonads at later stages, indicating their possible roles in late gonad development (Additional file 2: Figure S1). *Cdn15* was highly expressed in primary spermatocytes and secondary spermatocytes, as well as in the oocytes. *Rpl* was expressed in spermatogonia and efferent duct, and in the phase II oocytes of the ovary. In contrast, no signal was detected in either testis or ovary using the *Borealin*, *Gtsf1*, *ZarI*, *Cdn15* and *Rpl* sense probes. The fact that these novel genes recapitulate the expression patterns of the key sex genes used to identify relevant gene expression modules provides evidence for the biological relevance of these gene modules.

## Conclusions

In this study, we chose gonadal transcriptome data of XX and XY fish at 20, 30, 40, 90 and 180 dah, as well as 45 and 90 dat, to investigate the specific events that trigger sex determination and differentiation. We found that transcriptomic profiles of female and male gonads at 5 and 20 dah exhibited high similarities. Gene co-expression analysis constructed interactive networks of highly correlated genes that modulated sexual differentiation in XX and XY gonads. The global gene expression profiles during gonad development will provide early insights into the potential processes and pathways involved in sexual differentiation. We propose the present candidate genes as a starting point for future mathematical modeling and integration of more genes regarding the regulatory network of sex determination and sexual differentiation. The network can be upgraded later in several aspects, for example, incorporating additional nodes and interactions (i.e. details of TFBS), as well as modeling different cell lineages of the gonad such as the Leydig or theca cells. A functional analysis of the identified candidate genes is required to help elucidate their potential significance in gonadal sex determination and differentiation process.

## Additional files

Additional file 1: Candidate genes involved in tilapia sex determination and differentiation. (XLSX 65 kb)

Additional file 2: **Figure S1.** Cellular localization of *C15orf65* and *Rbp2* in tilapia testis and ovary by ISH. *C15orf65* was highly expressed in primary spermatocytes and secondary spermatocytes (A), as well as in the oocytes (B). *Rbp2* was expressed in spermatogonia and efferent duct (C), and in the phase II oocytes of the ovary (D). PSC, primary spermatocytes; SSC, secondary spermatocytes; OC, oocytes; I-IV, phase I to phase IV oocytes; SG, spermatogonia; ED, efferent duct; Arrowheads indicate the positive signal. (TIF 40771 kb)
