# Supplementary figures and images for: Transcriptome display during tilapia sex determination and differentiation as revealed by RNA-Seq analysis
Source: BMC Genomics. 2018 May 15;19:363. doi: 10.1186/s12864-018-4756-0 (PMC5952695; doi:10.1186/s12864-018-4756-0)

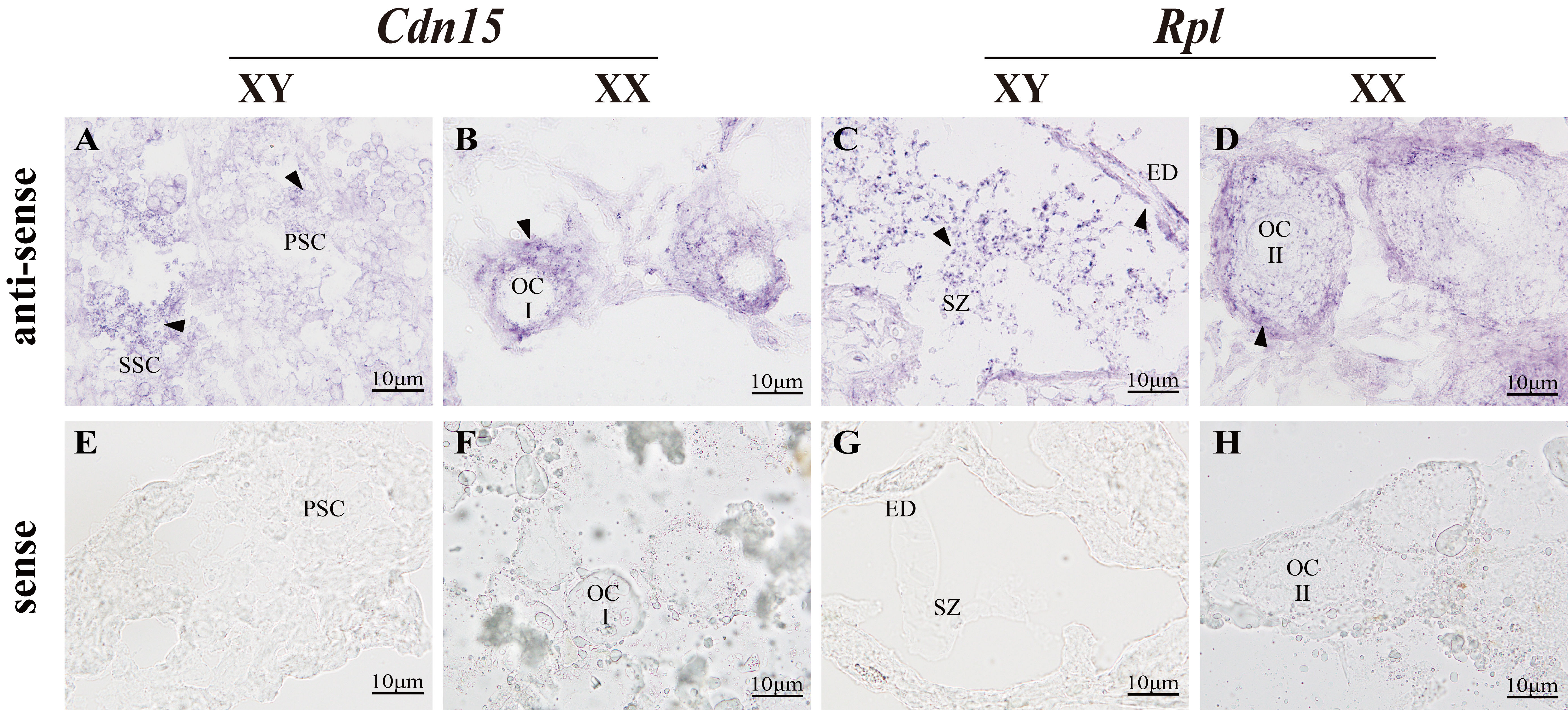

Supplement: Supplementary file 2 — Figure S1. Cellular localization of C15orf65 and Rbp2 in tilapia testis and ovary by ISH. C15orf65 was highly expressed in primary spermatocytes and secondary spermatocytes (A), as well as in the oocytes (B). Rbp2 was expressed in spermatogonia and efferent duct (C), and in the phase II oocytes of the ovary (D). PSC, primary spermatocytes; SSC, secondary spermatocytes; OC, oocytes; I-IV, phase I to phase IV oocytes; SG, spermatogonia; ED, efferent duct; Arrowheads indicate the positive signal. (TIF 40771 kb) [file 12864_2018_4756_MOESM2_ESM.tif]
